# Bayesian deconvolution for computational cognitive modeling of fMRI data

**DOI:** 10.1016/j.neuroimage.2025.121213

**Published:** 2025-04-11

**Authors:** Jonathon R. Howlett, Katia M. Harlé, Alan N. Simmons, Charles T. Taylor

**Affiliations:** a VA San Diego Healthcare System, San Diego, CA, USA; b Department of Psychiatry, University of California San Diego, La Jolla, CA, USA

**Keywords:** Computational psychiatry, Reward processing, Bayesian inference, Monetary incentive delay, Striatum, Reinforcement learning

## Abstract

A central goal of cognitive neuroscience is to make inferences about underlying cognitive processes from observable data. However, current fMRI analysis tools cannot directly estimate latent parameters in computational cognitive models from blood-oxygen-level-dependent (BOLD) signal. Here, we present a novel Bayesian deconvolution technique for full hierarchical generative cognitive modeling of fMRI timeseries data. We validated this approach by applying Bayesian deconvolution to the monetary incentive delay (MID) task to identify processes underlying incentive anticipation in a sample of 54 individuals who underwent 2 scan sessions as part of a clinical trial for anxiety and depression. Based on a series of Bayesian models, we found evidence that striatal reward region activity reflects incentive prediction error rather than raw incentive value during anticipation of monetary loss or gain. Test-retest analyses found that individual parameters estimated using a generative Bayesian learning model (including a persistent prior parameter and a *β* parameter representing a scaling term between prediction error and BOLD signal) were estimated more reliably than an index derived from traditional fMRI analysis (beta value for contrast between gain and no gain during anticipation). Our method holds potential for broad application to diverse neural processes and individual differences in health and disease.

## 1. Introduction

Advances in generative Bayesian computational modeling have recently enabled direct inference of latent cognitive parameters—hidden variables influencing observed behaviors—from behavioral data (Adams et al., 2016; Montague et al., 2012). However, generative Bayesian cognitive models have not yet been applied to fMRI data. A novel, fully Bayesian hierarchical approach to fMRI analysis would enable direct inference of latent parameters from BOLD timeseries, yielding posterior probability distributions on these parameters at the level of individual subjects. Beyond making inferences about latent parameters at the individual level, generative Bayesian modeling of fMRI data would have the additional advantage of enabling Bayesian model selection using techniques that balance model complexity against predictive accuracy (Vehtari et al., 2017). Prior work has applied Bayesian deconvolution to estimate neural activity levels underlying BOLD signal (Makni et al., 2008), but direct estimation of latent cognitive parameters in a fully hierarchical Bayesian model—a technique with potential for broad application in cognitive neuroscience research—remains an unmet challenge.

Here, we present a novel Bayesian deconvolution method which enables full hierarchical cognitive modeling for fMRI studies using a Markov-chain Monte-Carlo (MCMC) algorithm (Carpenter et al., 2017). MCMC estimates posterior probability distributions of latent model parameters (e.g. prior expectations in a learning model) based on observed data (e.g. BOLD signal), by iteratively drawing samples of model parameters (Homan and Gelman, 2014; Neal, 1993). To apply MCMC to a full hierarchical cognitive model with fMRI data, we perform a crucial step: executing separate convolutions with the hemodynamic response function (HRF) using the drawn parameter values in each MCMC iteration. In this way, we implement a fully Bayesian deconvolution on the entire probability distribution over regressors, given all possible combinations of parameters. This contrasts with the traditional approach, where frequentist deconvolution is performed using a single fixed regressor, using fixed parameter values typically set arbitrarily or fit based on behavioral data but not inferred from the BOLD signal itself (Gläscher and O’Doherty, 2010).

As an illustration and validation of our new technique, we applied the method to a new learning model of anticipatory incentive processing. Reward processing has often been investigated using computational learning models, based on the premise that a striatal reward region encodes reward prediction errors on reward receipt (O’Doherty et al., 2007). Two considerations suggest that incentive processing during reward anticipation may involve computationally sophisticated learning processes, similar to what has been observed during reward receipt. First, incentive processing drives motivated behavior (Knutson and Greer, 2008) and may result in allocation of effort toward the most rewarding tasks. Effort should be allocated based not only on the incentive available (raw incentive value), but should be modulated based on the background mean incentive rate of the environment, which must be learned from experience (Kurzban et al., 2013). Second, in order for a finite range of neural activity to efficiently convey a potentially large range of incentive values, the mapping between incentive values and firing rates should be adjusted adaptively based on the statistics of the environment (Kobayashi et al., 2010) (a view which is also supported by the broader predictive coding literature (de-Wit et al., 2010)). Again, the mean incentive rate of the environment must be learned from experience, so actual incentive can be conveyed efficiently as a deviation from this expected value. These considerations suggest that the brain must estimate the average incentive rate of the environment from experience and modulate neural activity accordingly. A previous study found that striatal activity was associated with the value of an incentive cue for a particular trial minus the average value of incentive cues across trials (Knutson and Wimmer, 2007), but the process by which average incentive value is updated dynamically has not been explored in detail. A simple means to accomplish this is a delta learning rule similar to Rescorla Wagner (RW) learning (Rescorla and Wagner, 1972), in which estimates of average incentive value are updated according to a prediction error when each incentive cue is encountered. Similar to reward receipt, model-based fMRI could be applied to examine the learning process which updates predictions about the mean incentive value during anticipatory incentive processing.

We applied our Bayesian modeling technique to fMRI data from the monetary incentive delay (MID) task (Knutson et al., 2001a; Kryza-Lacombe et al., 2021). We hypothesized that striatal reward region activation during incentive anticipation would reflect prediction error rather than raw incentive value as an efficient method to encode incentive signals. We modeled the learning process generating this trial-by-trial prediction error using RW and related variants. We then compared test-retest reliability of individual-level parameters fit using this process with individual indices estimated using traditional fMRI analysis (i.e., linear regression), based on the hypothesis that a Bayesian learning model would enable more reliable estimation of individual parameters compared to traditional techniques.

## 2. Materials and methods

### 2.1. Experimental design

The experiment was part of a larger study investigating the mechanistic effects of a positive valence targeted psychosocial intervention – Amplification of Positivity (AMP) – on social reward processing (ClinicalTrials.gov Identifier: NCT03196544). The study was a three-arm, parallel randomized controlled trial of two doses of AMP (5 and 10 sessions) vs. waitlist control. Participants were randomized to one of these three arms, and underwent assessments both prior to and after the intervention (or waitlist). In addition to the primary social reward task (data reported elsewhere (Taylor et al., 2023)), participants performed an fMRI monetary incentive delay (MID) task (Knutson et al., 2001a; Kryza-Lacombe et al., 2021) which is the focus of the current manuscript. Pre- and post-assessment MID sessions occurred approximately 10 weeks apart for all subjects regardless of condition.

### 2.2. Participants

The sample included individuals who endorsed clinically elevated symptoms of anxiety (Overall Anxiety Severity and Impairment Scale; OASIS ≥ 8 (Campbell-Sills et al., 2009)) or depression (Patient Health Questionnaire; PHQ-9 ≥ 10 (Kroenke et al., 2001)) and displayed evidence of social disconnection (Social Connectedness Scale Revised; SCSR < 90 (Lee et al., 2001)) as well as social functioning impairments (Sheehan Disability Scale – Social domain; SDS-Social ≥ 5 (Leon et al., 1997)). Individuals ages 18–55 who met these criteria were recruited through primary care clinics as well as posted announcements. Fifty-four participants completed both imaging sessions. Two participants were excluded for low (<90 %) fMRI signal-to-noise ratio and one participant was excluded for motion (greater than 15 % of timepoints censored for motion). The final sample was comprised of fifty-one participants (age: 30.96 ± 9.57; 18 male, 32 female, and 1 identified as neither male nor female; 18 received waitlist control, 20 received 5-session AMP, and 13 received 10-session AMP). Diagnostic interviews were conducted using the Mini International Neuropsychiatric Interview for DSM-5 (MINI Version 7.0.2 (Sheehan, 2016; Sheehan et al., 1998)). All study procedures were approved by the UCSD Institutional Review Board, and all participants provided written informed consent prior to participation. Data will be made available through the National Institute of Mental Health Data Archive (NDA) data repository.

### 2.3. Monetary incentive delay task

Participants performed an MID task (Knutson et al., 2001a; Kryza-Lacombe et al., 2021) during fMRI acquisition at baseline and post-treatment (or after a 10-week wait period). On each trial, participants were shown a cue indicating potential monetary gains or losses of $0.00, $1.00, or $5.00. Participants could either gain or avoid losing money by pressing a button while a target was presented. During an anticipation phase, a cue shape was presented, followed by a crosshair. Then, a white target was presented, during which participants were required to quickly respond via button press. The target was followed by a delay, followed by presentation of the outcome stating how much money participants had gained or lost for that trial. Trials were separated by a variable inter-trial interval of 2000, 4000, or 6000 ms.

### 2.4. fMRI data preprocessing and time series extraction

Participants were scanned in a 3T General Electric 750 scanner using an 8-channel head array coil. Each scanning session included a three-plane scout scan, a sagittally acquired spoiled gradient recalled (SPGR) sequence for acquiring T1-weighted images (172 slices; thickness: 1 mm; TI=450 ms, TR=8 ms, TE=3 ms; matrix: acquired 192×256; FOV=256 cm; flip angle=12 degrees; sagittal plane) and T2*-weighted axially acquired echo-planar imaging (EPI) scans to measure blood oxygen level dependent (BOLD) signals (parameters: slice thickness=3 mm; TR=2 s, TE=32 ms, flip angle=70 degrees; matrix=64×64, FOV=240 mm).

**Fig. 1.**
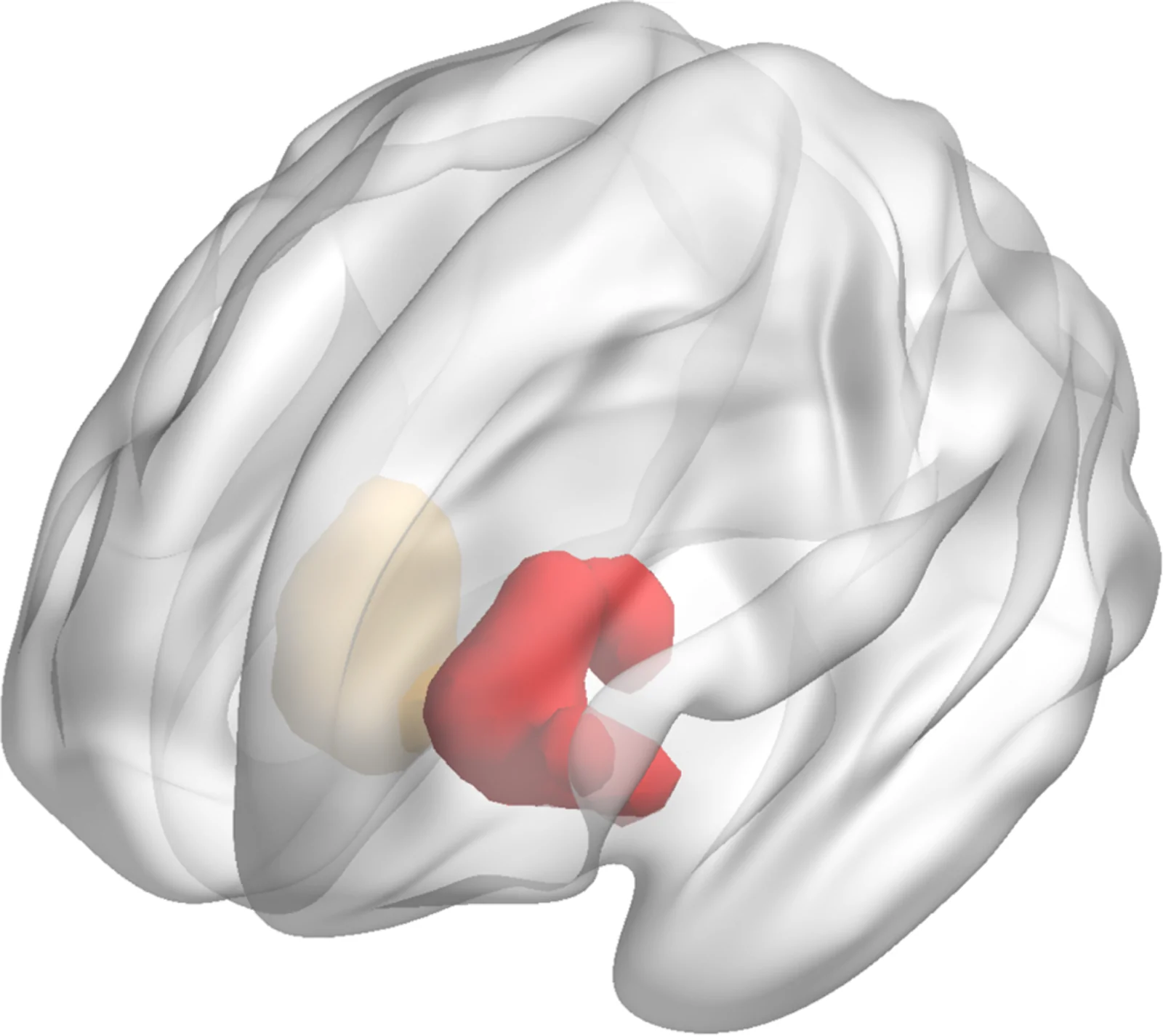
Striatal Reward Region. Blood oxygen level dependent (BOLD) signal was extracted bilaterally from a functionally defined striatal reward region of interest (ROI) based on Neurosynth. This BOLD timeseries was used in models examining incentive anticipation processing.

Imaging analyses were conducted using Analysis of Functional Images (AFNI) (Cox, 1996). Standard preprocessing steps were used with the afni.proc.py tool including removal of outlying acquisitions, despiking, slice time correction, co-registration of anatomical and functional scans, spatial smoothing (6-mm half maximum smoothing kernel) and warping to standardized MNI space. Data was then extracted for the striatal reward region based on NeuroSynth (Yarkoni et al., 2011), for optimal replication we used version 5 release of the 200 topic set, topic #63, we clustered this (AFNI: 3dclust) using contiguous faced voxels with a minimum z-score of 6 and cluster size of 100 contiguous voxels (800 mm^3^). This resulted in two striatal clusters placed bilaterally (10, 176 mm^3^, x/y/*z*=− 13.5/− 8.8/− 1.7 & 15,104 mm^3^, x/y/*z* = 14.5/− 5.3/− 1.6; see Fig. 1). This approach was taken to create an unbiased and replicable approach rather than to maximize signal.

The mean of the extracted timeseries from left and right striatal clusters was calculated. We therefore used a univariate timeseries in subsequent analyses. A linear regression was performed with BOLD percent signal change as dependent variable and predictors including nuisance regressors (head motion in the x, y, z, roll, pitch, and yaw directions and fourth-degree polynomials to model low-frequency drift) as well as 12 consumption phase regressors (2 (gain, loss) x 3 ($0, $1, $5) x 2 (hit, miss)) convolved with an HRF. The HRF was the AFNI canonical gamma distribution (GAM) function (an identical HRF was used for the Bayesian analyses described below). This single gamma function (Cohen, 1997) has been frequently used to model striatal HRF in previous studies (Greer et al., 2014; Knutson et al., 2001b; Sawe et al., 2022). See the Supplement for analyses with alternative HRFs. This regression yielded residualized values for each timepoint, representing BOLD signal controlling for nuisance regressors and consumption phase regressors. These residualized BOLD signals were used as the striatal reward region timeseries in subsequent analyses to examine the anticipation phase. Outlying volumes were censored in all analyses.

### 2.5. Preliminary fMRI analyses

All fMRI regression analyses were performed in Bayesian hierarchical fashion using the RStan interface of the Stan language (Stan Development Team, 2018). Each model assumed that BOLD percent signal change depends linearly on a set of regressors (different regressors were used for each model), with Gaussian noise. All regressors were constructed by convolving a vector of trial events (occurring during the anticipation phase) with an HRF, which was identical to the HRF used in AFNI in the traditional fMRI analyses. The anticipation response was time-locked to incentive cue presentations for all models, thus accounting for timing on the task. Individual model parameters included an intercept term, noise term (standard deviation of Gaussian noise), and beta weights (regression coefficients) representing scaling factors between the regressors and BOLD percent signal change. In turn, individual-level regression coefficients were modeled as being drawn from group-level Gaussian distributions in hierarchical fashion, allowing for estimation of posterior distributions on group mean parameters.

Prior to performing fMRI analyses using the Rescorla-Wagner learning model, we first performed a preliminary “model-free” analysis designed to test the hypothesis that striatal activity is influenced by incentive on the prior trial, consistent with a learning process in which striatal activity reflects a prediction error signal in which the prediction is updated trial-by-trial. We constructed a single, parametric incentive anticipation regressor which corresponded to the amount of money at stake, i.e. the amount that could be won or lost on each trial ($0, $1, and $5), occurring at cue presentation. We then constructed a second incentive anticipation regressor, identical to the first except lagged by one trial (i.e. representing the amount of money at stake on the previous trial). The first trial was discarded from analysis since there was no previous trial for the lagged regressor. This model was constructed to test the hypothesis that the current incentive would be positively associated with BOLD signal, while the previous incentive would be negatively associated with BOLD signal, consistent with a learning process in which expectations are updated on each trial and BOLD signal is associated with prediction error.

### 2.6. Rescorla-Wagner learning model

We constructed a Rescorla-Wagner (RW) (Rescorla and Wagner, 1972; Sutton and Barto, 1998) learning model in which individuals predict the incentive value of each trial based on the incentive value of previous trials. The expected incentive value, $V_{i}$ was updated by an amount equal to the prediction error (i.e., current incentive $R_{i}$ minus the previous $V_{i}$), scaled by a fixed learning rate which was set to 0.5:

$$V_{t+1}\;=\;V_{t}\;+\;\alpha \left(R_{t}\;-\;V_{t}\right).$$

At the start of the task, was initialized to 2, i.e. the mean value of the three incentive conditions (0, 1, and 5). The RW model was implemented in R (R Core Team, 2013) and generated a set of prediction errors for each trial. Since the order of trials by condition was pseudo-randomized and was the same for all subjects, the prediction error values for each trial were also the same across subjects.

### 2.7. Rescorla-Wagner fMRI analyses

To determine the relationship between RW prediction error and BOLD signal in the striatal reward region, we constructed a set of regressors representing trials binned by prediction error. We first placed each of the 90 trials into 10 bins of 9 trials each, in order of RW prediction error (most negative to most positive). We then constructed 10 regressors representing these ten bins, with each regressor including 9 trials with similar RW prediction error (active during the anticipation phase). This model was designed to test the hypothesis that trials with more negative prediction errors would be associated with more negative changes in BOLD signal, while those with more positive prediction error would be associated with more positive changes in BOLD signal. In order to quantify the relationship between prediction error and striatal reward region activation across bins (which was predicted to be linear), we calculated the Pearson correlation across bins between the mean prediction error within each bin and the median of the posterior distribution of the striatal reward region percent BOLD signal change for each bin.

### 2.8. Specificity of striatal response

In order to assess the specificity of incentive prediction error in the striatum compared to other regions, we additionally extracted signal from an anatomically defined auditory processing (superior temporal gyrus, posterior division) ROI. BOLD timeseries was extracted from the superior temporal gyrus, posterior division, based on the Harvard-Oxford atlas (Desikan et al., 2006). We then created a Bayesian hierarchical regression model in which RW prediction error, convolved with the HRF, was the dependent variable and striatal reward region BOLD signal and superior temporal gyrus BOLD signal were the two predictors.

### 2.9. Individual learning models

We compared two models of the learning process with individual parameters: an individual learning rate model and an individual persistent prior model. For the individual learning rate model, BOLD signal was modeled as depending on prediction errors generated by an RW model:

$$PE_{t}\;=\;R_{t}\;-\;V_{t}$$

$$V_{t+1}\;=\;V_{t}\;+\;\alpha \;*\;PE_{t}.$$

For each session, the learning rate α, constrained to the interval (0, 1), was modeled as an inverse logit transformation of an individual-level $\alpha_{raw}$ parameter. This parameter was modeled in hierarchical fashion as being drawn from a distribution with a group-level mean and standard deviation.

**Fig. 2.**
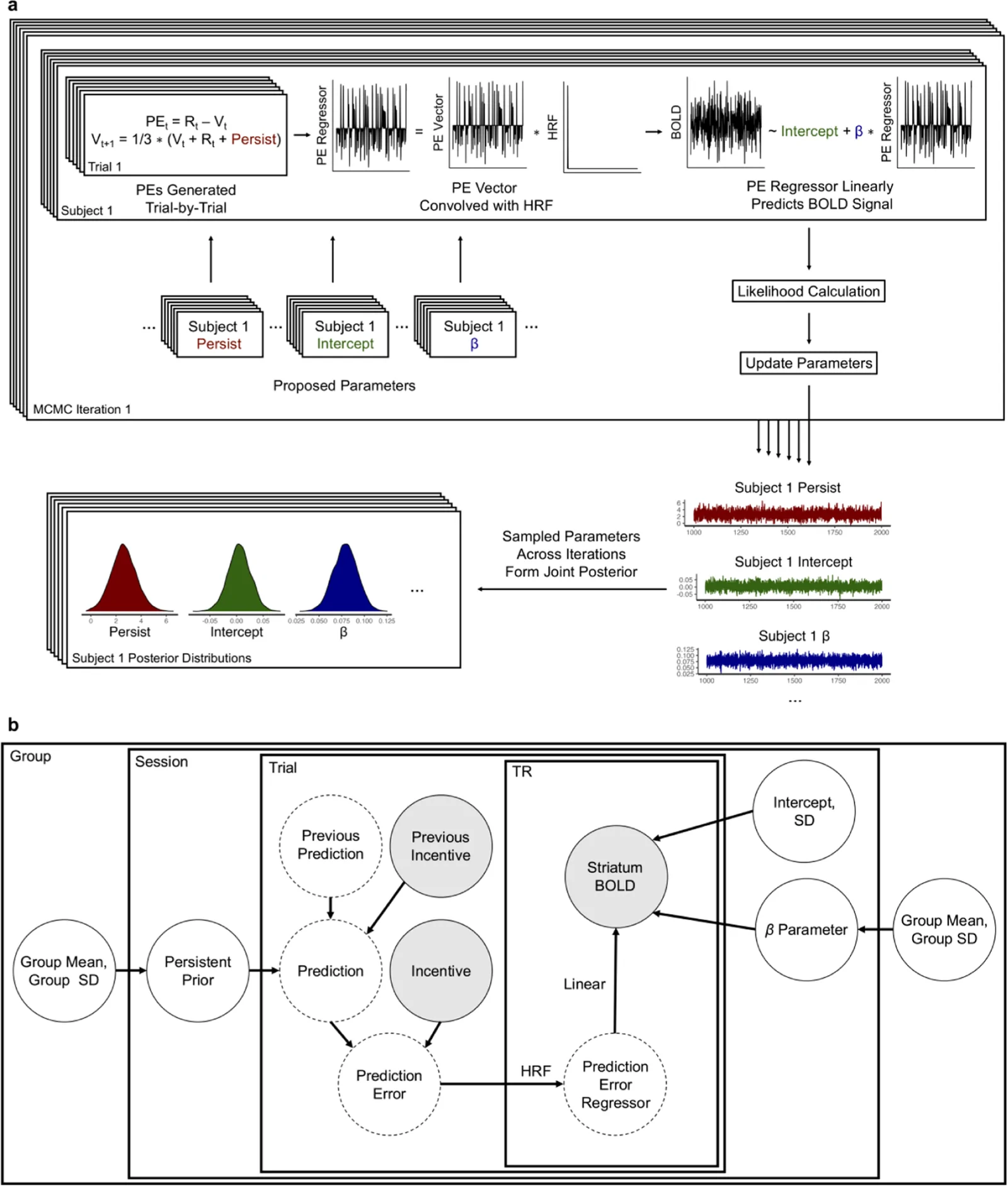
Bayesian Deconvolution Procedure. a. Parameter estimation via Markov chain Monte Carlo (MCMC). For each MCMC iteration, a set of parameter values are proposed. These are used to generate a vector of prediction errors (PEs) over trials using the learning model. PEs are then convolved with the hemodynamic response function (HRF) to generate a PE regressor. BOLD signal is modeled as being linearly dependent on this regressor. Likelihood calculations are used to generate the next proposal for parameter values, while the current parameter values are stored, comprising the sampled posterior distribution across iterations. b. Graphical depiction of individual persistent prior model. Striatal reward region BOLD timeseries was used to estimate individual parameters including a persistent prior term via Bayesian inference. In the forward model, an incentive prediction is generated on each trial by combining the previous prediction, previous incentive, and persistent prior term. The prediction error is then convolved with the hemodynamic response function (HRF) to generate a prediction error regressor. Striatal reward region BOLD signal is modeled as being linearly dependent on this regressor, with a *β* parameter that scales the relationship between prediction error and BOLD signal. The persistent prior parameter and *β* parameter were modeled in Bayesian hierarchical fashion as being drawn from group level distributions.

For the individual persistent prior model, prediction errors were modeled as being generated via a modified RW learning process, in which each prediction was generated by mixing the previous prediction, the current incentive, and a persistent prior expectation (see Fig 2):

$$PE_{t}\;=\;R_{t}\;-\;V_{t}$$

$$V_{t+1}\;=\;1/3\;*\;V_{t}\;+\;1/3\;*\;R_{t}\;+\;1/3\;*\;prior.$$

This persistent prior effect has previously been demonstrated in human learning (Ryali et al., 2018). Note that, to aid in convergence, the three mixing factors were fixed at 1/3 rather than using two additional free parameters (with the third factor being 1 minus the first two) as in a generic persistent prior model. At the start of the task, $V_{1}$ was initialized to the persistent prior value. The persistent prior term was modeled as an individual parameter in Bayesian hierarchical fashion, depending on a group-level mean and standard deviation. In both models, the vector of prediction errors across trials was convolved with the HRF for each subject within each Markov-chain Monte Carlo (MCMC) draw of the parameters to generate a prediction error regressor, $Reg_{PE}$. The BOLD timeseries was modeled as depending on this regressor in linear fashion:

$$BOLD\;∼\;Normal\left(Intercept\;+\;\beta \;*\;Reg_{PE,}\;\sigma \right).$$

### 2.10. Model comparison

In order to compare the two learning models, we first performed a model comparison using leave-one-out cross-validation (Vehtari et al., 2017). We then performed simulations and parameter recovery with each model to assess how accurately the individual parameters could be estimated. BOLD timeseries data was generated for 100 simulated sessions for both the individual learning rate model and the individual persistent prior model. Raw learning rate parameters (for the individual learning rate model) and persistent prior parameters (for the individual persistent prior model), as well as *β* parameters (for both models) were sampled from normal distributions with means and standard deviations taken from the group level distributions estimated in Stan from the empirical data. Simulated learning rates were generated by applying the logit transform to the raw learning rate parameters. Other parameters (intercept terms and standard deviation terms) were sampled without replacement from empirical subject values estimated in Stan. Prediction errors were generated for each trial (via the RW and persistent prior models), then convolved with the HRF to generate prediction error regressors. BOLD values were generated by sampling from a normal distribution centered on the simulated prediction error regressors. Parameter recovery was performed via MCMC using the simulated BOLD timeseries data, exactly as performed for the empirical data. We assessed recovery of the learning rate and persistent prior parameters as well as the *β* parameter (i.e. the scaling factor between prediction error and neural activation).

### 2.11. Test-Retest reliability

We performed test-retest reliability measurements for parameters from the preferred model based on model comparison (the individual persistent prior learning model). For each individual experimental session, we extracted the individual estimates of the persistent prior parameter as well as for *β*, the scaling factor between the prediction error and striatal reward region BOLD signal (the median of the posterior distribution was used as a point estimate for each session). We then computed intraclass correlation coefficients (ICC based on a single fixed rater model) for the persistent prior parameter and for *β* across session 1 and session 2 for the median using R (R Core Team, 2013).

For comparison purposes, we also computed test-retest reliability for an MID index derived from traditional fMRI analysis (beta value for anticipation gain vs. no gain contrast in the striatal reward region). This contrast was selected because it has been commonly used in past studies (Elliott et al., 2020) and because the *β* parameter from the Bayesian analysis represents a difference in activations across trials, analogous to a contrast between trial types, rather than activations in a set of trials compared to baseline.

## 3. Results

### 3.1. Preliminary fMRI analyses

For gain trials, an incentive of $5 was associated with greater BOLD percent signal change in the striatal reward region (median *β* = 0.084, 95 % credible interval *β* = [0.057, 0.111]) compared with an incentive of $0 (median *β* = − 0.074, 95 % credible interval *β* = [− 0.095, − 0.052]; value for $5 greater than for $0 in 100 % of posterior draws) and an incentive of $1 (median *β* = − 0.022, 95 % credible interval *β* = [− 0.042, − 0.003]; value for $5 greater than for $1 in 100 % of posterior draws). Similarly, for loss trials, an incentive of $5 was associated with greater BOLD percent signal change in the striatal reward region (median *β* = 0.009, 95 % credible interval *β* = [− 0.011, 0.030]) compared with an incentive of $0 (median *β* = − 0.080, 95 % credible interval *β* = [− 0.099, − 0.063]; value for $5 greater than for $0 in 100 % of posterior draws) and an incentive of $1 (median *β* = − 0.080, 95 % credible interval *β* = [− 0.096, − 0.064]; value for $5 greater than for $1 in 100 % of posterior draws).

**Fig. 3.**
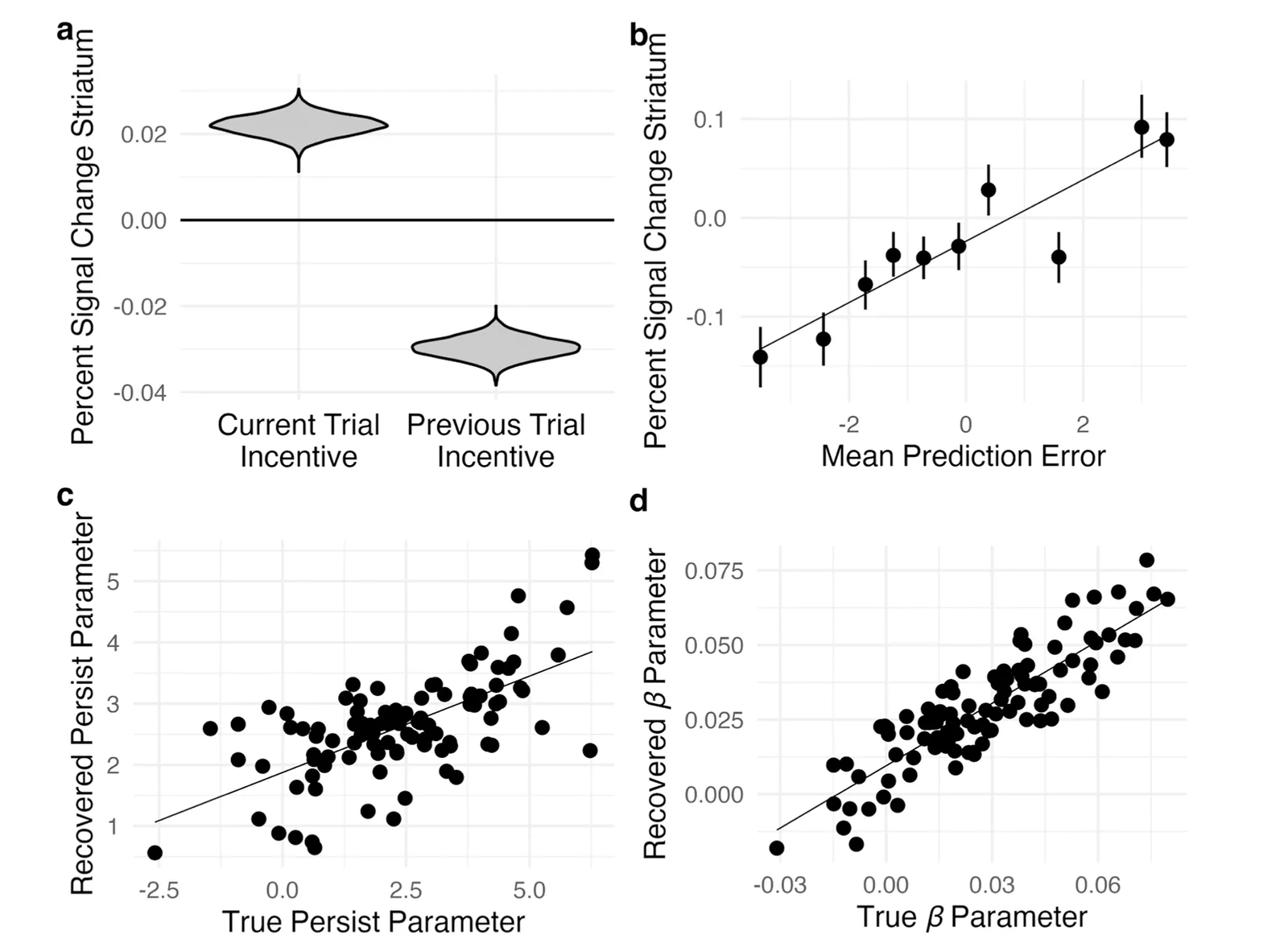
Results of Bayesian Models of Incentive Anticipation in the Striatum. a. Violin plot showing Bayesian posterior distributions of striatal reward region percent signal change associated with incentive value for the current trial and incentive value for the previous trial during the anticipation phase. Current trial incentive was associated with increased striatal reward region activity, while previous trial incentive was associated with decreased striatal reward region activity, consistent with an account in which the striatal reward region reflects incentive prediction error rather than raw incentive value. b. Scatterplot showing relationship between mean prediction error and striatal reward region percent signal change in the anticipation phase in binned trials. Trials were placed into 10 bins of 9 trials each, in order of Rescorla-Wagner (RW) prediction error (most negative to most positive). Black circles show the median of the Bayesian posterior distribution and bars show the 95 % credible interval. c. Scatterplot showing true and recovered persistent prior (persist) parameter for simulated data for 100 subjects. Parameters for simulations were selected based on estimated ranges from empirical data. d. Scatterplot showing true and recovered *β* parameter (scaling factor between prediction error and BOLD signal) for simulated data for 100 subjects.

As predicted, the incentive anticipation regressor was positively associated with BOLD percent signal change in the striatal reward region (median *β* = 0.009, 95 % credible interval *β* = [0.005, 0.012]; 100 % of the posterior greater than zero). When the incentive of the previous trial was included in the model, current trial incentive was *positively* associated with BOLD percent signal change in the striatal reward region (median *β* = 0.022, 95 % credible interval *β* = [0.017, 0.027]; 100 % of the posterior greater than zero), while previous trial incentive was *negatively* associated with BOLD percent signal change in the striatal reward region (median *β* = − 0.030, 95 % credible interval *β* = [− 0.034, − 0.025]; 100 % of the posterior less than zero), consistent with the hypothesis that striatal reward region activation encodes a prediction error based on a prediction which is updated trial-by-trial (see Fig. 3a).

### 3.2. Rescorla-Wagner fMRI analyses

In the model in which trials were placed into 10 bins in order of RW prediction error, the mean prediction error for each bin was linearly associated with striatal reward region BOLD percent signal change associated with each bin (*r* = 0.92, *p <* .001; see Fig. 3b). Similarly, when the incentive for each trial was also included in the model, mean prediction error for each bin continued to be linearly associated with striatal reward region BOLD percent signal change associated with each bin (*r* = 0.89, *p <* .001).

As predicted, RW prediction error was positively associated with BOLD percent signal change in the striatal reward region during anticipation (median *β* = 0.029, 95 % credible interval *β* = [0.023, 0.034]; 100 % of the posterior greater than zero). RW prediction error continued to be positively associated with BOLD percent signal change in the striatal reward region during anticipation (median *β* = 0.013, 95 % credible interval *β* = [0.005, 0.020]; 99.9 % of the posterior greater than zero) after controlling for current incentive (median *β* = 0.011, 95 % credible interval *β* = [0.005, 0.017]; 100 % of the posterior greater than zero) and previous incentive (median *β* = − 0.021, 95 % credible interval *β* = [− 0.027, − 0.016]; 100 % of the posterior less than zero).

### 3.3. Specificity of striatal response

Striatal BOLD signal, controlling for superior temporal gyrus BOLD signal, remained positively associated with RW prediction error regressor, convolved with the HRF (median *β* = 0.48, 95 % credible interval *β* = [0.40, 0.56]; 100 % of the posterior greater than zero). Superior temporal gyrus, controlling for striatal BOLD signal, was instead negatively associated with RW prediction error, convolved with the HRF (median *β* = − 0.20, 95 % credible interval *β* = [− 0.26, − 0.14]; 100 % of the posterior less than zero). This may be because the auditory processing region is relatively suppressed on high incentive trials on the primarily visual MID task. The results support the specificity of incentive prediction error response in the striatal reward region. See the Supplement for additional analyses with the superior temporal gyrus ROI.

### 3.4. Learning model comparison

Model comparison favored the individual persistent prior learning model (leave-one-out information criterion (LOOIC)=46,875.8, standard error (SE)=674.4) over the individual learning rate model (LOOIC=47,036.2, SE=675.8), but the difference in LOOIC estimates was within the standard error of the estimates.

For the individual persistent prior model, the correlation between the actual and recovered persistent prior parameter was *r* = 0.65 (*p* < .001; Fig 3c) and the correlation between the actual and recovered *β* parameter was *r* = 0.87 (*p* < .001; Fig 3d). For the individual learning rate model, simulations and parameter recovery found that the correlation between the actual and recovered learning rate parameter was *r* = 0.45 (*p* < .001) and the correlation between the actual and recovered *β* parameter was *r* = 0.85 (*p* < .001).

### 3.5. Test-Retest reliability

For the persistent prior parameter in the full sample, ICC was 0.26 (*p* = .03). For the *β* parameter, ICC was 0.47 (*p* < .001).

For the MID index derived from traditional fMRI analysis techniques (beta value for anticipation gain vs. no gain contrast in the striatal reward region) in the full sample, the ICC was 0.13 (*p* = .18).

Within the waitlist group (*n* = 18), for the persistent prior parameter, ICC was 0.36 (*p* = .07). For the *β* parameter, ICC was 0.31 (*p* = .10). For beta value for anticipation gain vs. no gain contrast in the striatal reward region, ICC was 0.29 (*p* = .11).

## 4. Discussion

In this study, we applied a novel Bayesian deconvolution technique to examine individual differences in anticipatory incentive processing in a functionally defined striatal reward region. We found robust evidence that striatal reward region activation during incentive processing reflects a prediction error in incentive value, rather than raw incentive value. Furthermore, we found that estimates of two parameters in the learning process (encoding a persistent prior expectation of incentive and a scaling factor between prediction error and BOLD signal) were correlated between the two fMRI sessions (pre- and post-intervention), with ICC estimates of 0.26 and 0.47, respectively. The incentive prediction error response was specific to the striatum compared to a superior temporal gyrus control region and was robust to different choices of HRF. Taken together, these results suggest that a novel approach toward model-based fMRI using full hierarchical Bayesian inference can measure meaningful individual variability in incentive processing.

Our ICC estimate of 0.47 for the *β* parameter falls at the higher end of estimated ICC values across a range of fMRI task effects, which is 0.067–0.485 based on a recent review (Elliott et al., 2020). It is important to note that some estimates of ICC values for reward anticipation in the striatum are also at the higher end of this range (Elliott et al., 2020; Wu et al., 2014). It should also be noted that the ROI selection was founded on maximizing replication and minimizing bias rather than maximizing robustness (i.e., ICC), thus it should not be inferred that the current report reflects the maximal ICC for the Bayesian or traditional analysis and that the current report ICCs should not be contrasted with reports that took alternative approaches. Importantly, analyses restricted to the waitlist control group (given concern that treatment effects in the AMP groups might reduce ICC) found a higher ICC estimate for the traditional analysis compared to the ICC estimated from the full sample, but the waitlist subsample was underpowered to obtain a reliable ICC estimate. It will therefore be important to repeat these analyses in a larger sample without treatment arms in the future to determine more accurate ICC values. However, the good performance of the Bayesian approach compared the traditional approach in the current study suggests that the Bayesian approach has the potential to improve reliability of parameter estimates.

The current work is novel in its use of Bayesian inference to estimate parameters in cognitive models from fMRI data. Previous work has applied Bayesian inference to fMRI analysis, such as using spatial priors to perform smoothing in order to regularize voxel-wise timeseries signals (Penny et al., 2005). Other work has applied MCMC specifically to fMRI data, including for mapping visual receptive fields (Adaszewski et al., 2018; Carvalho et al., 2020) and visual connective fields (Invernizzi et al., 2022). MCMC has also been applied to spatial smoothing of fMRI data (de Pasquale et al., 2008) and to hierarchical clustering of dynamic causal models (Yao and Stephan, 2021). While there have been several applications of Bayesian inference and MCMC to fMRI data, to our knowledge this study represents the first application of a Bayesian technique to infer parameters in a computational cognitive model directly from BOLD signal. While computational cognitive models have been widely incorporated into fMRI studies, previous approaches have either fixed model parameters at the group level or estimated model parameters based on behavior before generating model-based regressors, and then used traditional techniques to examine associations between these regressors and BOLD signal (Wilson and Niv, 2015). In contrast, Bayesian inference enables inverting a forward model in order to make inferences about cognitive parameters directly from BOLD response. The present results thus represent a novel application of MCMC which complements previous work on Bayesian inference in fMRI.

An important aspect of our method in the current study was a focus on temporal, rather than spatial, aspects of fMRI data. Much effort has previously been devoted to developing techniques leveraging the spatial component of fMRI data to localize processing in specific brain regions. Here, we took a different approach, applying Bayesian model-based analysis to the temporal rather than spatial aspect of fMRI. Since the striatum is a clear a priori region of interest for incentive processing, and the MID task is a robust probe of striatal activity (Knutson and Greer, 2008), the BOLD time series extracted from this region is ideal for use in model-based analyses. However, a similar approach could be applied to neural processes without an a priori region of interest, by using a data-driven decomposition such as independent component analysis (ICA) (McKeown et al., 2003) to derive timeseries which can then be used in Bayesian model-based analyses.

The flexibility of full generative Bayesian model-based fMRI could lend itself to multiple future extensions to examine incentive processing and other cognitive processes. MCMC, as implemented in Stan, enables highly flexible Bayesian inference applicable to a large range of problems (Carpenter et al., 2017). MCMC will take a forward model specifying the expected distribution of data for a given parameter value, along with actual data, and invert the model to produce a posterior distribution over parameter values. Stan can also be used for model comparison (Vehtari et al., 2017), which can be used to select between competing hypotheses for how cognitive processes are implemented in neural circuits. MCMC, as implemented in Stan, is also ideal for incorporating multiple data sources (i.e. BOLD signal, behavior, and other signals such as physiology or EEG) and for creating hierarchical models (in which sources of variability at the group and individual level are modeled simultaneously) (McGlothlin and Viele, 2018). Bayesian hierarchical approaches have proven advantageous in cognitive modeling for their ability to properly account for different sources of variation, combine different cognitive processes and different types of data into unified models, and integrate distinct cognitive models (Lee, 2011).

Rather than measuring neural responses to different task conditions, generative Bayesian model-based analysis requires explicit specification of an underlying cognitive process which generates these responses. In the current study, the approach involved modeling an adaptive learning process which is not apparent based on traditional fMRI analysis. As illustrated by our results, appropriate model specification with parameters that can be recovered given the data is critical to derive statistically reliable indices of individual variability in the underlying process. Based on our data, a persistent prior parameter was able to be more reliably recovered than a learning rate parameter, consistent with previous research showing that a range of learning rates tend to predict similar data distributions (and that it is therefore difficult to determine learning rate with precision based on the data) (Wilson and Niv, 2015). The *β* parameter, which represents scaling between prediction error and neural activation, was the most reliably estimated, and exhibited higher test-retest reliability than indices derived from traditional fMRI analyses. The results illustrate the importance of constructing and validating models that enable reliable estimation of individual parameters which may be useful in assessments of neural function.

Reinforcement learning models have previously been applied to reward receipt in the MID (with learning occurring about the probability of success on each trial) (Cao et al., 2019). This previous approach used standard fMRI analysis techniques (i.e. linear regression) with fixed computational parameters (rather than using generative Bayesian modeling to estimate computational parameters based on BOLD signal). To our knowledge, the current study represents the first application of Bayesian model-based fMRI to anticipatory incentive processing. This distinction between incentive anticipation and receipt highlights a gap between the large empirical literature on reward anticipation and traditional computational modeling of reward learning, which tends to focus on prediction errors upon reward receipt. The striatum is robustly activated by incentive anticipation (Knutson et al., 2001a) and is involved in regulating the vigor of both appetitive and aversively motivated behavior (Floresco, 2015). It has been hypothesized that altered striatal function may underlie psychiatric disorders which involve disruption of motivated behavior (Goto and Grace, 2008). The present results indicate that incentive anticipation in a striatal reward region is sensitive to changes in expectations which are updated by recent experience. This is consistent with optimal foraging accounts in which effort or vigor is modulated based on the background mean incentive rate of the environment (Kurzban et al., 2013). Our results suggest that Bayesian deconvolution may be a useful approach to measuring this learning process. Future research can examine the potential clinical implications of disrupted learning processes underlying incentive anticipation in the striatum.

This study has several limitations. Given the small effect sizes typically observed for relationships between self-report scales and indices derived from fMRI, the study was under-powered to detect these relationships robustly. Future work can examine these relationships by applying the model to larger samples. Another limitation was that the sample was heterogeneous in terms of intervention; one-third-of participants were placed on a wait list, while two-thirds received the AMP intervention. A treatment effect may therefore have influenced results, potentially by reducing apparent test-retest reliability. However, the waitlist control subsample was too small to obtain reliable ICC estimates. Additionally, the presence of mental health symptoms in the sample may have influenced the results. To obtain more reliable ICC estimates, it will be important to perform similar analyses in a larger sample without an intervention that may affect reliability and in a healthy control sample. Another limitation was that the high computational demands of this analysis limited us to an ROI approach; future work could extend the methods used here to timeseries from data-driven decompositions, multiple ROIs, or whole brain approaches. However, given the strong evidence for the striatum as an ROI in the MID, and the novelty of the analyses, an ROI approach was selected for this initial test of our Bayesian deconvolution approach. It is also important to note that our use of the term “learning” follows the convention from the cognitive science literature in which learning (e.g. in standard reinforcement learning paradigms(Niv, 2009)) can involve moment-to-moment adaptations in internal representations or behavior based on information from the environment. This learning process may represent a rapid tracking of estimates of environmental variables (e.g. environmental reward rates) rather than information storage in long-term memory. Future research is necessary to further investigate the underlying biological mechanisms of these learning processes. Additionally, we used one specific HRF (gamma function), but future research can also employ alternative HRF shapes, which may improve measurement reliability.

In conclusion, we developed a new technique for Bayesian deconvolution for computational cognitive models in fMRI studies. This novel method not only allows direct estimation of individual latent cognitive parameters from BOLD signal (which may be useful for studying individual differences in neural computation) but also enables hierarchical cognitive modeling (accounting for different sources of variation and incorporating multiple processes into unified models) and Bayesian model comparison to select between different competing models of computational processes instantiated in neural circuits (which can be used to test broader theories about neural computation at the group level). This advance can therefore enhance the utility of fMRI data to improve understanding of the neural implementation of cognitive processes at both the group and individual level. Our method could be widely applied in cognitive neuroscience studies to support either individual measurement of neurocomputational processes or selection between competing computational models, although future work is needed to assess this possibility. Our method holds potential for wide application in cognitive neuroscience studies, supporting both individual measurement of neurocomputational processes as well a selection between competing theories of neural computation.

## Supplementary Material

1

Supplementary material associated with this article can be found, in the online version, at doi:10.1016/j.neuroimage.2025.121213.

## Data Availability

Data will be made available through the National Institute of Mental Health Data Archive (NDA). Code is available at DOI 10.5281/zenodo.15091508; https://github.com/jonhowlett/bayesian-deconvolution
