# Supplementary material for: Bayesian deconvolution for computational cognitive modeling of fMRI data
Source: Neuroimage. Author manuscript; Available in PMC 2026 Aug 20. (PMC13492560; doi:10.1016/j.neuroimage.2025.121213)
Supplement: Supplementary file 1 [file NIHMS2174420-supplement-1.docx]

**Supplementary Methods**

**Monetary Incentive Delay Task**

Participants performed a monetary incentive delay (MID) task (Knutson et al., 2001; Kryza-Lacombe et al., 2021) during fMRI acquisition at baseline and post-treatment (or after a 10-week wait period). On each trial, participants were shown a cue indicating potential monetary gains or losses of $0.00, $1.00, or $5.00. Participants could either gain or avoid losing money by pressing a button while a target was presented. There were six task conditions comprised of gain and loss trials of $0.00, $1.00, or $5.00. Each condition was repeated 15 times each in pseudo-randomized order (with the order being the same for all participants) for a total of 90 trials. Each trial consisted of a cue, an anticipation phase, target presentation, and outcome presentation (see Supplementary Fig. 1). During the anticipation phase (4000 ms) one of six cue shapes was presented (2000 ms), followed by a crosshair (2000 ms). Then, a white target was presented, during which participants were required to quickly respond via button press. Target duration varied and was set to 250 ms at the start of the task, then titrated such that participants succeeded on approximately 66% of target responses. The target was followed by a delay (2000 ms minus duration of target presentation for any given trial), followed by presentation of the outcome (2000 ms) stating how much money participants had gained or lost for that trial. Trials were separated by a variable inter-trial interval of 2000, 4000, or 6000 ms.

**Hierarchical Bayesian Regression for Arbitrary Regressors**

Code was developed using the Stan (Carpenter et al., 2017) language to perform Bayesian hierarchical regression for a set of regressors specified at the trial level. First, each regressor at the trial level was convolved with an arbitrary hemodynamic response function (HRF) vector to produce regressors at the repetition time (TR) level. For each regressor *reg_i_,* the regression coefficient (slope term) was fit in a hierarchical manner. Each individual-level coefficient term *β_n,i_* (i.e. the coefficient for the *reg_i_* for participant *n*) was modeled as being drawn from a Gaussian group distribution with a group mean *μ_β,i_* and standard deviation *σ_β,i_*:

*β_n,i_* ~ *normal*(*μ_β,i_*, *σ_β,i_*).

Then, BOLD response was modeled as linearly depending on an individual-level intercept as well as the set of regressors:

*BOLD_n_* ~ normal(*intercept_n_* + Σ *β_n,i_* * *reg_i_*, *σ_n_*),

where *σ_n_* is a individual-level standard deviation. Censored volumes were removed from this regression. Weakly informative prior distributions were used, with *μ_i_* and *intercept_n_* having Gaussian priors with mean 0 and standard deviation 10 and *σ_n_* having a half-normal prior with mean 0 and standard deviation 5.

**Individual Learning Models**

See the main text for equations for the learning models. For the individual learning rate model, the learning rate parameter was modeled as an inverse logit transformation of an individual-level *α_raw_* parameter. This parameter was modeled in hierarchical fashion as being drawn from a Gaussian distribution with a group-level mean and standard deviation:

*α_n,raw_* ~ *normal*(*μ_α_*, *σ_α_*).

The prior for *μ_α_* was a Gaussian distribution with mean 0 and standard deviation 20, while the prior for *σ_α_* was a half-normal distribution with mean 0 and standard deviation 10. For the persistent prior model, the individual-level *Prior* parameter was modeled in hierarchical fashion as being drawn from a group-level Gaussian distribution:

*Prior_n_* ~ normal(*μ_Prior_*, *σ_Prior_*).

The prior for *μ_Prior_* was a Gaussian distribution with mean 0 and standard deviation 20, while the prior for *σ_Prior_* was a half-normal distribution with mean 0 and standard deviation 10.

In both individual learning models, the individual-level slope terms *β_n,PE_* were modeled in hierarchical fashion as being drawn from group-level Gaussian distributions:

*β_n,PE_* ~ *normal*(*μ_β_*, *σ_β_*).

In both models, BOLD response was modeled as depending linearly on an individual-level intercept *intercept_n_* and on prediction error *PE* (according to the learning model):

*BOLD_n_* ~ normal(*intercept_n_* + *β_n,PE_* * *PE_n_*, *σ_n_*).

In both models, the prior on *μ_β_* as well as *intercept_n_* was a Gaussian distribution with mean 0 and standard deviation 10. The prior on *σ_β_* as well as *σ_n_* was a half-normal distribution with mean 0 and standard deviation 5. All model-fitting was performed using default RStan settings for MCMC sampling with 4 Markov chains.

**Nucleus Accumbens ROI Analyses**

We performed additional analyses using signal extracted from an anatomically defined nucleus accumbens (NAcc) ROI. BOLD timeseries was extracted from left and right NAcc based on the Harvard-Oxford atlas (Desikan et al., 2006). Left and right NAcc timeseries were averaged together to generate a bilateral NAcc timeseries. Processing was otherwise identical to analyses described in the main text. We applied the model in which trials were placed into 10 bins in order of RW prediction and these ten bins served as regressors (see main text for more details), with the hypothesis that mean prediction error for each bin would be linearly associated with NAcc BOLD percent signal change, as was true for the functional striatal reward region.

**Auditory Processing ROI Analyses**

As a negative control analysis, to determine whether our findings regarding incentive prediction error were specific to reward processing regions, we also performed analyses using signal extracted from an anatomically defined auditory processing (superior temporal gyrus, posterior division) ROI. BOLD timeseries was extracted from the superior temporal gyrus, posterior division, based on the Harvard-Oxford atlas (Desikan et al., 2006). Processing was otherwise identical to analyses described in the main text. We applied the model in which trials were placed into 10 bins in order of RW prediction and these ten bins served as regressors (see main text for more details), with the hypothesis that mean prediction error for each bin would not be associated with superior temporal gyrus BOLD percent signal change, unlike the functional striatal reward region.

**Alternative HRF Analyses**

To ensure that results did not depend on the specific choice of HRF, we performed additional analyses with two alternative HRF models. The first alternative was the canonical HRF used in statistical parametric mapping (SPM) (Friston et al., 1994). Unlike the single gamma function used in the main analyses, this HRF is a double gamma function, i.e. a linear combination of two gamma functions (Lindquist et al., 2009). The second alternative HRF was empirically derived directly from the data by taking the mean residualized BOLD signal across all participants over the first 9 TR timepoints starting from the first trial (the first trial was chosen because response was not influenced by prior trials). For both of these alternative HRFs, the model which included current trial incentive and previous trial incentive as regressors, as well as the model in which trials were placed into 10 bins in order of RW prediction error were run.

**Supplementary Results**

**MID Behavioral Analysis**

The overall hit rate for the MID task, averaged across subjects, was 0.56. The hit rates for all conditions were between 0.5 and 0.6 (0.53 for Lose $0, 0.54 for Lose $1, 0.57 for Lose $5, 0.57 for Win $0, 0.58 for Win $1, and 0.57 for Win $5).

**Collinearity of Lagged Incentive Regressors**

The Pearson correlation between current trial incentive value and previous trial incentive value at the trial level was r = .08 (p = .45). The Pearson correlation between the current trial incentive regressor and the previous trial incentive regressor at the TR level was r = .36 (p < .001).

**Nucleus Accumbens ROI Analyses**

In the model in which trials were placed into 10 bins in order of RW prediction error, the mean prediction error for each bin was linearly associated with NAcc BOLD percent signal change associated with each bin (r = .82, p = .004; see Supplementary Fig. 2).

**Auditory ROI Analyses**

In the model in which trials were placed into 10 bins in order of RW prediction error, the mean prediction error for each bin was not significantly associated with auditory ROI BOLD percent signal change associated with each bin (r = .34, p = .34; see Supplementary Fig. 3).

**Alternative HRF Analyses**

For the double gamma function HRF, current trial incentive was positively associated with BOLD percent signal change in the striatal reward region (median *β* = 0.14, 95% credible interval *β* = [0.12, 0.17]; 100% of the posterior greater than zero), while previous trial incentive was negatively associated with BOLD percent signal change in the striatal reward region (median *β* = -0.13, 95% credible interval *β* = [-0.15, -0.11]; 100% of the posterior less than zero), consistent with the main analyses (see Supplementary Fig. 4a). In the model in which trials were placed into 10 bins in order of RW prediction error, the mean prediction error for each bin was linearly associated with striatal reward region BOLD percent signal change associated with each bin (r = .94, p < .001; see Supplementary Fig. 4b).

For the empirically estimated HRF, current trial incentive was positively associated with BOLD percent signal change in the striatal reward region (median *β* = 0.10, 95% credible interval *β* = [0.09, 0.12]; 100% of the posterior greater than zero), while previous trial incentive was negatively associated with BOLD percent signal change in the striatal reward region (median *β* = -0.05, 95% credible interval *β* = [-0.06, -0.04]; 100% of the posterior less than zero), consistent with the main analyses (see Supplementary Fig. 4c). In the model in which trials were placed into 10 bins in order of RW prediction error, the mean prediction error for each bin was linearly associated with striatal reward region BOLD percent signal change associated with each bin (r = .92, p = .001; see Supplementary Fig. 4d).

**References**

Carpenter, B., Gelman, A., Hoffman, M. D., Lee, D., Goodrich, B., Betancourt, M., Brubaker, M., Guo, J., Li, P., & Riddell, A. (2017). Stan: A Probabilistic Programming Language. *Journal of Statistical Software*, Medium: ED; Size: 32 p. <https://www.osti.gov/servlets/purl/1430202>

Desikan, R. S., Segonne, F., Fischl, B., Quinn, B. T., Dickerson, B. C., Blacker, D., Buckner, R. L., Dale, A. M., Maguire, R. P., Hyman, B. T., Albert, M. S., & Killiany, R. J. (2006, Jul 1). An automated labeling system for subdividing the human cerebral cortex on MRI scans into gyral based regions of interest. *Neuroimage, 31*(3), 968-980. <https://doi.org/10.1016/j.neuroimage.2006.01.021>

Friston, K. J., Jezzard, P., & Turner, R. (1994). Analysis of functional MRI time-series. *Human Brain Mapping, 1*(2), 153-171. <https://doi.org/https://doi.org/10.1002/hbm.460010207>

Knutson, B., Adams, C. M., Fong, G. W., & Hommer, D. (2001). Anticipation of increasing monetary reward selectively recruits nucleus accumbens. *J.Neurosci., 21*(16), 159-164. PM:11459880 (NOT IN FILE)

Kryza-Lacombe, M., Pearson, N., Lyubomirsky, S., Stein, M. B., Wiggins, J. L., & Taylor, C. T. (2021, Jul). Changes in neural reward processing following Amplification of Positivity treatment for depression and anxiety: Preliminary findings from a randomized waitlist controlled trial. *Behav Res Ther, 142*, 103860. <https://doi.org/10.1016/j.brat.2021.103860>

Lindquist, M. A., Meng Loh, J., Atlas, L. Y., & Wager, T. D. (2009, Mar). Modeling the hemodynamic response function in fMRI: efficiency, bias and mis-modeling. *Neuroimage, 45*(1 Suppl), S187-198. <https://doi.org/10.1016/j.neuroimage.2008.10.065>

**Supplementary Figure 1**

Supplementary Figure 1: Monetary Incentive Delay Task.

Each trial consists of a cue, anticipation phase, target presentation, and outcome presentation.

**Supplementary Figure 2**

Supplementary Figure 2: Incentive Anticipation in the Nucleus Accumbens.

Scatterplot showing relationship between mean prediction error and percent signal change in the anticipation phase in binned trials in the anatomically defined NAcc ROI. Trials were placed into 10 bins of 9 trials each, in order of Rescorla-Wagner (RW) prediction error (most negative to most positive). Black circles show the median of the Bayesian posterior distribution and bars show the 95% credible interval.

**Supplementary Figure 3**

Supplementary Figure 3: Incentive Anticipation in an Auditory Processing Region (Superior Temporal Gyrus, Posterior Division).

Scatterplot showing relationship between mean prediction error and superior temporal gyrus, posterior division percent signal change in the anticipation phase in binned trials. Trials were placed into 10 bins of 9 trials each, in order of Rescorla-Wagner (RW) prediction error (most negative to most positive). Black circles show the median of the Bayesian posterior distribution and bars show the 95% credible interval.

**Supplementary Figure 4**

Supplementary Figure 4: Alternative Hemodynamic Response Function (HRF) Analyses.

a. Violin plot showing Bayesian posterior distributions of striatal reward region percent signal change associated with incentive value for the current trial and incentive value for the previous trial during the anticipation phase, using the double gamma function HRF. Current trial incentive was associated with increased striatal reward region activity, while previous trial incentive was associated with decreased striatal reward region activity, consistent with the main analyses.

b. Scatterplot showing relationship between mean prediction error and striatal reward region percent signal change in the anticipation phase in binned trials, using the double gamma function HRF. Trials were placed into 10 bins of 9 trials each, in order of Rescorla-Wagner (RW) prediction error (most negative to most positive). Black circles show the median of the Bayesian posterior distribution and bars show the 95% credible interval.

c. Violin plot showing Bayesian posterior distributions of striatal reward region percent signal change associated with incentive value for the current trial and incentive value for the previous trial during the anticipation phase, using the empirically estimated HRF. Current trial incentive was associated with increased striatal reward region activity, while previous trial incentive was associated with decreased striatal reward region activity, consistent with the main analyses.

d. Scatterplot showing relationship between mean prediction error and striatal reward region percent signal change in the anticipation phase in binned trials, using the empirically estimated HRF. Trials were placed into 10 bins of 9 trials each, in order of Rescorla-Wagner (RW) prediction error (most negative to most positive). Black circles show the median of the Bayesian posterior distribution and bars show the 95% credible interval.
